# Comprehensive microRNA profiling reveals potential augmentation of the IL1 pathway in rheumatic heart valve disease

**DOI:** 10.1186/s12872-018-0788-2

**Published:** 2018-03-16

**Authors:** Qiyu Lu, Yi Sun, Yuyin Duan, Bin Li, Jianming Xia, Songhua Yu, Guimin Zhang

**Affiliations:** 1 Department of Gastrointestinal Surgery, The Second Affiliated Hospital of Kunming Medical University, Kunming, Yunnan Province 650101 China; 2 Department of Cardiothoracic Surgery, The First Affiliated Hospital of Kunming Medical University, Kunming, Yunnan Province 650101 China

**Keywords:** Rheumatic heart disease, Valvular heart disease, miRNA, Interleukin 1, Inflammation

## Abstract

**Background:**

Valvular heart disease is a leading cause of cardiovascular mortality, especially in China. More than a half of valvular heart diseases are caused by acute rheumatic fever. microRNA is involved in many physiological and pathological processes. However, the miRNA profile of the rheumatic valvular heart disease is unknown. This research is to discuss microRNAs and their target gene pathways involved in rheumatic heart valve disease.

**Methods:**

Serum miRNA from one healthy individual and four rheumatic heart disease patients were sequenced. Specific differentially expressed miRNAs were quantified by Q-PCR in 40 patients, with 20 low-to-moderate rheumatic mitral valve stenosis patients and 20 severe mitral valve stenosis patients. The target relationship between certain miRNA and predicted target genes were analysis by Luciferase reporter assay. The IL-1β and IL1R1 expression levels were analyzed by immunohistochemistry and western blot in the mitral valve from surgery of mitral valve replacement.

**Results:**

The results showed that 13 and 91 miRNAs were commonly upregulated or downregulated in all four patients. Nine miRNAs, 1 upregulated and 8 downregulated, that had a similar fold change in all 4 patients were selected for quantitative PCR verification. The results showed similar results from miRNA sequencing. Within these 9 tested miRNAs, hsa-miR-205-3p and hsa-miR-3909 showed a low degree of dispersion between the members of each group. Hsa miR-205-3p and hsa-miR-3909 were predicted to target the 3’UTR of IL-1β and IL1R1 respectively. This was verified by luciferase reporter assays. Immunohistochemistry and Western blot results showed that the mitral valve from rheumatic valve heart disease showed higher levels of IL- 1β and IL1R1 expression compared with congenital heart valve disease. This suggested a difference between rheumatic heart valve disease and other types of heart valve diseases, with more inflammatory responses in the former.

**Conclusion:**

In the present study, by next generation sequencing of miRNAs, it was revealed that interleukin 1β and interleukin 1 receptor 1 was involved in rheumatic heart diseases. And this is useful for diagnosis and understanding of mechanism of rheumatic heart disease.

**Electronic supplementary material:**

The online version of this article (10.1186/s12872-018-0788-2) contains supplementary material, which is available to authorized users.

## Background

Valvular heart disease (VHD) is a major leading cause of cardiovascular mortality. According to the most recent statistics, the prevalence of any VHD in the entire U.S. population is 2.7%, with 0.4% being aortic stenosis, 0.5% aortic regurgitation, 0.1% mitral stenosis, and 1.7% mitral regurgitation [1]. According to a five-year epidemiological survey in the Guangdong Cardiovascular Institute, there were 69.7% patients with a relatively definite aetiology for VHD. Though the prevalence of rheumatic heart disease (RHD) decreased during the past ten years, it still accounted for 37% of VHD patients. Mitral stenosis and mitral regurgitation account for a large proportion of heart valve disease in China because of the prevalence of acute rheumatic fever two to three decades ago [2].

MicroRNAs are the best characterized non-coding RNA family. They have a fundamental importance in normal development, differentiation and growth control and in human diseases [3]. Though the roles of miRNAs in many physiological and pathological processes have been widely and deeply investigated, the roles of miRNAs in valvular heart disease have just begun to be studied [1]. The investigation of miRNA profile changes in valvular heart disease will benefit the diagnosis and treatment of diseases. On the one hand, miRNAs are potentially involved in the regulation of key VHD-related pathways, such as cell cycle control, inflammation and fibrosis. On the other hand, due to their role in VHD, miRNA molecules could become diagnostic and prognostic biomarkers for patient stratification and therapeutic targets and agents [4].

Rheumatic heart disease (RHD) is the most common acquired heart disease in children in many countries of the world, especially in developing countries, including China. Though the global burden of disease caused by rheumatic fever is currently decreasing, it is still responsible for approximately 233,000 deaths annually. Up to 1% of all schoolchildren in Africa, Asia, the Eastern Mediterranean region, and Latin America show signs of the disease (these data were cited from the website of the World Heart Federation). Additionally, some individuals who suffered from rheumatic fever 10 to 30 years ago survived with heart valve damage and still need healthcare today. Thus, in-depth investigation of the mechanism of rheumatic valvular heart disease is urgent for the understanding and treatment of the disease. In the present study, we assessed the miRNA expression profile in rheumatic valvular heart disease patients and revealed a role for the interleukin 1 pathway in this disease.

## Methods

### Patients

Eighty subjects from the First Affiliated Hospital of Kunming Medical University (Kunming, China), between April 2015 and December 2017, were selected for the present study. All subjects collected from 30 to 65 years old individuals. Of these subjects, 40 were RHD patients, 20 were congenital valvular heart disease patients, and the remaining 20 were normal healthy adults with no medical history of congenital heart disease, cardiomyopathy, liver or renal diseases. The inclusion criteria, as described in [4], for the RHD group are as follows: (i) every patient diagnosed with mitral valve prolapse because of mitral chordae tendineae fracture and mitral insufficiency and scheduled for mitral valve replacement; (ii) left ventricular ejection fraction (EF) > 50%; and (iii) left ventricular end-diastolic diameter (LVEDD) < 55 mm. All human materials were obtained in accordance with the hospital’s regulations and hence were approved by the Ethics Committee of Kunming Medical University. Written informed consent was also obtained from all the subjects in advance.

### Tissue and serum collection

Human heart valve tissue samples were obtained from the patients who received mitral valve replacement surgery in the First Affiliated Hospital of Kunming Medical University. The tissues and serum were stored in liquid nitrogen until use.

### MiRNA sequencing

Total RNA, including miRNA, was extracted using Trizol Reagent (Invitrogen, Carlsbad, CA, USA) from serum and passed the RNA quality control for sequencing [5]. The quality and integrity of the total RNA were assessed with an Agilent 2100 Bioanalyzer (Agilent Technologies, USA). High-throughput next-generation sequencing was carried out to achieve optimal serum miRNA profiles. We carried out miRNA sequencing (Illumina, BGI, Shenzhen) following the manufacturer’s instructions. We screened the high quality clean read sequences by alignment to NCBI GenBank data and miRBase 21.0 for further analysis. We calculated the fold change (FC) and *P*-value via the t-test and corrected the P-value in the false discovery rate (FDR) using the Benjamini and Hochberg method. FDR ≤ 0.05 and | log2FC ≥ 2 were set as the cut-offs to screen out the differentially expressed miRNAs. The coordinately regulated miRNAs were selected for further investigation as described in the Results.

### Quantitative reverse transcription–PCR analysis of miRNA expression levels

Quantitative reverse transcription-PCR was used to validate the sequencing results [6]. Total RNA from serum, including miRNA, was extracted using Trizol Reagent (Invitrogen, CA). Reverse transcription of the total RNA was performed using an All-in-One First-Strand cDNA Synthesis kit (GeneCopoeia Inc., USA) according to the manufacturer’s protocol. Real-time PCR was performed by using All-in-OneTM qPCR Mix (Applied GeneCopoeia Inc., USA) on a Roche Lightcycler 480 System. U6 snRNA was used as the miRNA endogenous control. All samples were normalized to the internal control, and fold changes were calculated through relative quantification.

### Bioinformatics analysis

Four software programs (miRWalk, miRanda, RNA22 and Targetscan) were used for target gene prediction, and only the genes identified by all four approaches were selected [7]. To understand the possible functions of the predicted target genes, Gene Ontology (GO) [8] and Kyoto Encyclopedia of Genes and Genomes (KEGG) enrichment analyses were performed with the GO stats package (http://www.geneontology.org/), with a *P* value < 0.05 set as the cut-off to select significantly enriched terms.

### Luciferase reporter assay

The 3’UTR of IL1β was synthesized starting with the sequence “GAGCCTAGTT TTTAAT” to the end of the mRNA and cloned into the pmiRGLO vector [9]. HEK293 cells were cultured on a 12-well plate and transfected with 100 ng of the reporter plasmid, hsa-miR-205-3p mimic (200 nM in each well) or both. Firefly and Renilla activities were determined 24 h after transfection using Beetlejuice and Renilla juice reagents (Beyotime, China). The influences of hsa-miR-3909 on the IL1R1 3’UTR were tested in the same way. The 3’UTR of IL1R1 was cloned using the following primers: forward, 5′ CGATTGCAGGACACAAGCAC 3′ and reverse, 5′ AAGCAGGTGGAAAGGCAAGA 3′.

### Immunohistochemistry

Mitral valve tissues were fixed in 4% PFA for 24 h. After fixation, the tissue was dehydrated to enable embedding in paraffin, which is water insoluble. The tissue was dehydrated gently by immersion in increasing concentrations of alcohol. The alcohol was then cleared by incubation in xylene prior to paraffin embedding. The paraffin was typically heated to 60 °C and allowed to harden overnight. Finally, the tissue was sectioned into 8-μm-thick paraffin sections using a microtome. Sections were deparaffinized and rehydrated. Endogenous peroxidase activity was blocked by incubation for 30 min in 3% H_2_O_2_ in methanol at room temperature. Antigen retrieval was performed using microwave treatment for 15 min in citrate buffer (pH 6.0). The sections were blocked with 10% goat serum at 37 °C for 1 h and incubated with rabbit polyclonal antibody against human IL-1β or rabbit monoclonal antibody against human IL1RA (both applied at 1:200, Abcam, USA) in a humidified chamber overnight at 4 °C. Next, the sections were incubated with HRP conjugated secondary antibody for 1 h at room temperature, developed with DAB chromogen for 10 min at room temperature, rinsed in running tap water for 5 min, and counterstained with haematoxylin-eosin staining. The antibodies used in the present study are as follows: anti-IL1β antibody (ab2105, Abcam, USA) and anti-IL1R1 antibody (ab106278, Abcam, USA).

### Immunoblotting

Mitral valve tissues samples were lysed with RIPA Lysis Buffer (Beyotime, China) containing PMSF protease inhibitor (Beyotime, China). Tissue lysates were cleared by centrifugation at 12,000×*g* for 10 min. Tissue lysates (20–30 μg protein) were separated on a 10% SDS–polyacrylamide gel and transferred onto PVDF membranes. The membrane was blocked in Tris-buffered saline (20 mM Tris, 150 mM NaCl, pH 7.4) containing 5% BSA and 0.1% Tween-20 for 2 h and then incubated with primary antibodies (1:1000 dilution) overnight. After blocking, washing, and incubation with appropriate secondary antibody, blots were developed using Super Signal chemiluminescence reagents (Pierce, Rockford, IL, USA). The bands of interest from the immunoblots were scanned by densitometry, and the integrated density of the pixels in the identified areas was quantified using ImageJ (NIH, Bethesda, MD). The antibodies used in the present study were as follows: anti-IL1 beta antibody (ab2105, Abcam, USA), anti-IL1R1 antibody (ab106278, Abcam, USA), β-Actin (8H10D10) mouse mAb antibody (#3700, Cell Signaling Technology, USA), goat anti-mouse IgG-HRP antibody (sc-2005, Santa Cruz Technology, USA) and goat anti-rabbit IgG-HRP (sc-2004, Santa Cruz, USA).

## Results

### Expression profiling of plasma miRNAs by miRNA sequencing

**Fig. 1:**
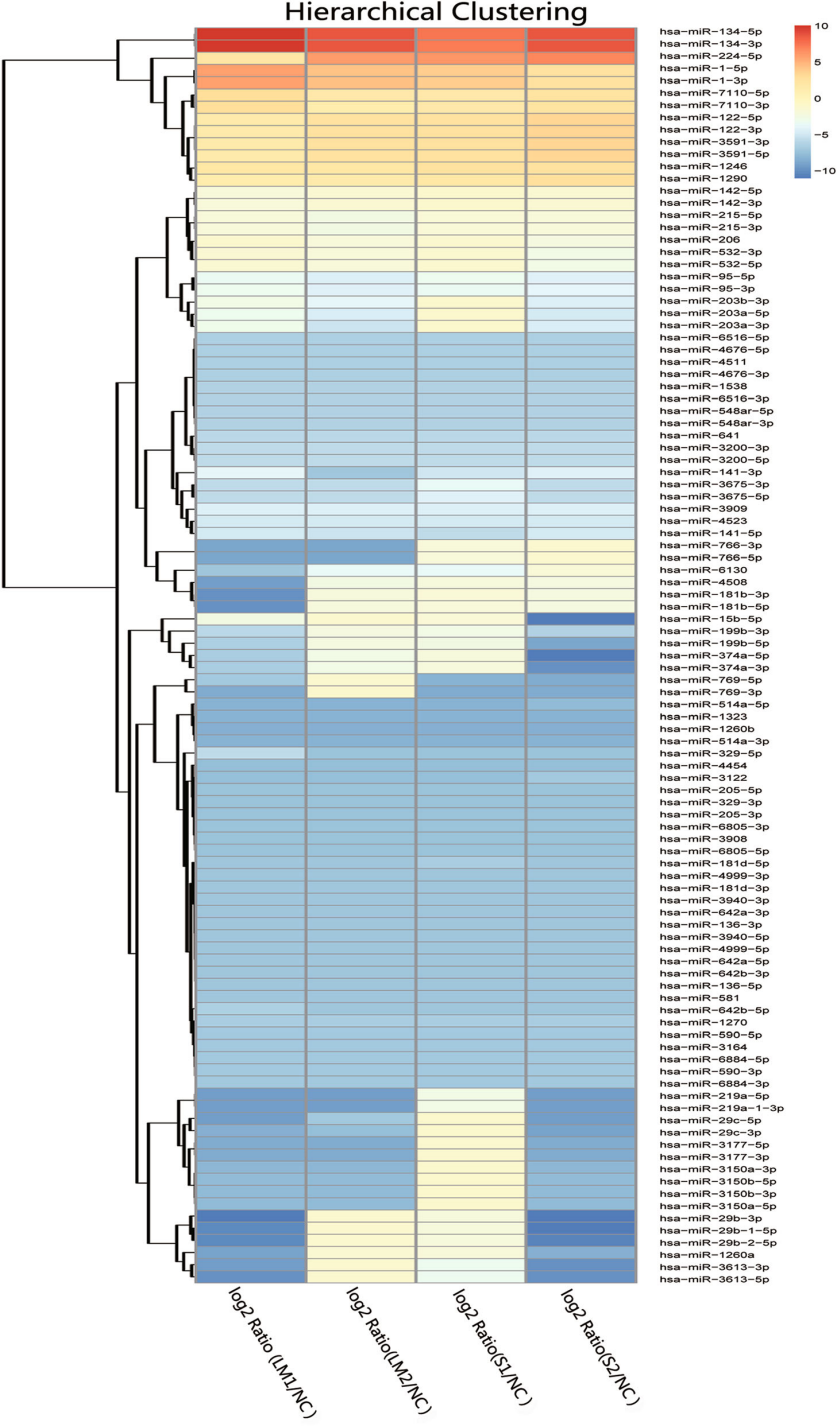
Heat map of miRNA expression levels in the serum of healthy individual and rheumatic heart valvular disease patients. The serum miRNA profile of one healthy individual (NC) and four rheumatic heart valvular disease patients, including 2 low-to-moderate mitral stenosis patients (LM1 and LM2) and 2 severe mitral stenosis patients (S1 and S2), were assayed by next generation sequencing. The coordinately expressed miRNAs are listed in the map

The miRNA of the serum from 4 patients who suffered rheumatic heart disease, 2 of whom (LM1 and LM2) had mild-to-moderate mitral stenosis and 2 of whom (S1 and S2) had severe mitral stenosis, were sequenced. miRNA in the serum from 1 healthy individual (NC) was also sequenced as a negative control. The miRNAs of the LM1, LM2, S1 and S2 patients were compared with those of healthy individuals. More than 200 miRNAs were downregulated and more than 20 miRNAs were over-expressed more than 2-fold in the patients’ serum (Additional file 1: Table S1; Additional file 2: Table S2; Additional file 3: Table S3 and Additional file 4: Table S4). However, more than one half of the down-expressed and over-expressed miRNAs between the patients were different. miRNAs that were commonly upregulated or downregulated in all four patients were selected and are shown in Table 1. The heat map of the differentially expressed miRNAs based on Table 1 is shown in Fig. 1. There were 13 upregulated and 91 downregulated genes.

**Table 1:** Coordinately regulated miRNAs in RHD patients with mitral valve stenosis and their fold of change

miRNA Name	log2Ratio(LM1/NC)	log2Ratio(LM2/NC)	log2Ratio(S1/NC)	log2Ratio(S2/NC)
hsa-miR-15b-3p	−2.3981	−1.1215	− 1.6477	− 11.3676
hsa-miR-29b-3p	− 11.0347	− 1.2671	−2.0974	− 11.0347
hsa-miR-29b-1-5p	− 10.2868	− 1.2696	− 2.1042	− 11.0238
hsa-miR-15b-5p	− 2.4033	−1.1285	− 1.6580	−10.9747
hsa-miR-374a-5p	− 6.7934	− 2.6911	−2.1515	− 10.9111
hsa-miR-29b-2-5p	−10.2882	− 1.2743	− 2.1146	− 10.4946
hsa-miR-374a-3p	− 6.7395	− 2.6759	−2.1522	− 9.9063
hsa-miR-3613-3p	− 9.1652	−1.2656	− 3.0141	− 9.9022
hsa-miR-3613-5p	− 9.8992	−1.2626	− 3.0677	− 9.8992
hsa-miR-219a-5p	− 9.2885	− 9.2885	−2.7493	− 9.2885
hsa-miR-219a-1-3p	− 9.2862	− 9.2862	−2.7713	−9.2862
hsa-miR-29c-5p	−9.1384	− 6.8165	− 1.0305	− 9.1384
hsa-miR-29c-3p	−8.3756	− 7.5276	− 1.0688	− 9.1126
hsa-miR-199b-5p	− 6.5267	− 2.1762	− 2.5637	−8.7706
hsa-miR-769-5p	− 6.8790	−1.1259	−8.0854	− 8.6159
hsa-miR-769-3p	−8.5862	−1.0881	− 8.5862	− 8.5862
hsa-miR-3177-5p	− 8.5175	− 8.5175	−1.2611	−8.5175
hsa-miR-3177-3p	−8.5016	−8.5016	−1.2172	− 8.5016
hsa-miR-1260a	−8.9245	−1.3404	−1.8813	− 8.3940
hsa-miR-1260b	−8.2931	− 8.2931	−8.2931	− 8.2931
hsa-miR-514a-3p	−8.2368	− 8.2368	−8.2368	− 8.2368
hsa-miR-1323	−8.1384	− 8.1384	−8.1384	− 8.1384
hsa-miR-3150a-3p	− 7.8704	− 7.8704	−1.0774	− 7.8704
hsa-miR-3150b-5p	− 7.8642	− 7.8642	− 1.0712	− 7.8642
hsa-miR-3150b-3p	− 7.8580	− 7.8580	−1.0265	− 7.8580
hsa-miR-3150a-5p	− 7.8517	− 7.8517	−1.0007	− 7.8517
hsa-miR-514a-5p	−8.2416	− 8.2416	− 8.2416	− 7.7111
hsa-miR-4454	−7.5715	− 7.5715	− 7.5715	−7.5715
hsa-miR-3908	−7.4197	−7.4197	− 7.4197	−7.4197
hsa-miR-6805-5p	−7.4113	−7.4113	− 7.4113	−7.4113
hsa-miR-6805-3p	−7.3856	−7.3856	− 7.3856	−7.3856
hsa-miR-205-5p	−7.3249	−7.3249	− 7.3249	−7.3249
hsa-miR-329-3p	−7.3068	−7.3068	− 7.3068	−7.3068
hsa-miR-205-3p	−7.2977	−7.2977	− 7.2977	−7.2977
hsa-miR-329-5p	−5.5607	−7.2977	−7.2977	− 7.2977
hsa-miR-181d-5p	−7.1832	− 7.1832	− 6.6527	−7.1832
hsa-miR-181d-3p	−7.1733	−7.1733	− 7.1733	−7.1733
hsa-miR-3940-3p	−7.1733	−7.1733	− 7.1733	−7.1733
hsa-miR-136-3p	−7.1632	−7.1632	− 7.1632	−7.1632
hsa-miR-3940-5p	−7.1632	−7.1632	− 7.1632	−7.1632
hsa-miR-642a-3p	−7.1632	−7.1632	− 7.1632	− 7.1632
hsa-miR-136-5p	− 7.1531	−7.1531	− 7.1531	−7.1531
hsa-miR-581	−7.1531	−7.1531	− 7.1531	−7.1531
hsa-miR-642b-3p	−7.1531	−7.1531	− 7.1531	−7.1531
hsa-miR-642b-5p	−6.4162	−7.1531	−7.1531	− 7.1531
hsa-miR-642a-5p	−7.1441	− 7.1441	−7.1441	− 7.1441
hsa-miR-4999-5p	−7.1339	− 7.1339	−7.1339	− 7.1339
hsa-miR-4999-3p	−7.1132	− 7.1132	−7.1132	− 7.1132
hsa-miR-590-5p	−6.9937	− 6.9937	− 6.9937	− 6.9937
hsa-miR-3122	− 7.5096	− 7.5096	− 7.5096	−6.9790
hsa-miR-3164	−6.9242	−6.9242	− 6.9242	−6.9242
hsa-miR-590-3p	−6.9002	−6.9002	− 6.9002	−6.9002
hsa-miR-6884-3p	−6.9002	−6.9002	− 6.9002	−6.9002
hsa-miR-6884-5p	−6.8881	−6.8881	− 6.8881	−6.8881
hsa-miR-1270	−6.7755	−6.7755	− 6.7755	−6.7755
hsa-miR-4676-5p	−6.4374	−6.4374	− 6.4374	−6.4374
hsa-miR-4511	−6.4207	−6.4207	− 6.4207	−6.4207
hsa-miR-4676-3p	−6.4207	−6.4207	− 6.4207	−6.4207
hsa-miR-6516-5p	−6.4037	−6.4037	− 6.4037	−6.4037
hsa-miR-199b-3p	−5.9433	−2.1902	− 2.6096	−6.3637
hsa-miR-1538	−6.3517	−6.3517	− 6.3517	−6.3517
hsa-miR-6516-3p	−6.3517	−6.3517	− 6.3517	−6.3517
hsa-miR-548ar-5p	−6.3159	−6.3159	− 6.3159	−6.3159
hsa-miR-548ar-3p	−6.2977	−6.2977	− 6.2977	−6.2977
hsa-miR-641	−5.9255	−5.9255	− 5.9255	− 5.9255
hsa-miR-3675-3p	− 5.7489	−5.7489	−3.5969	− 5.7489
hsa-miR-3675-5p	−5.7219	− 5.7219	−4.1369	−5.7219
hsa-miR-3200-3p	−5.5783	−5.5783	− 5.5783	−5.5783
hsa-miR-3200-5p	−5.5478	−5.5478	− 5.5478	−5.5478
hsa-miR-4523	−4.9015	−4.9015	− 4.9015	−4.9015
hsa-miR-203a-5p	−3.2221	−4.5280	−1.0208	−4.8728
hsa-miR-141-5p	−4.8613	−5.1832	−5.5983	−4.7093
hsa-miR-203a-3p	−2.9480	−5.1373	− 1.1112	−4.6747
hsa-miR-3909	−4.3847	−4.3847	− 4.3847	−4.3847
hsa-miR-203b-3p	−2.7659	−3.8329	−1.0359	−4.3682
hsa-miR-141-3p	−3.6781	−7.2224	−5.0704	−4.1635
hsa-miR-95-5p	−3.4150	−4.3479	−3.0525	−4.1520
hsa-miR-95-3p	−3.1872	−4.0941	−3.3392	− 3.8653
hsa-miR-532-3p	−1.5264	− 1.8552	− 1.1227	−2.7619
hsa-miR-532-5p	−1.5236	− 1.8643	−1.1235	−2.7513
hsa-miR-206	−1.0095	−1.9317	− 1.3186	−2.2835
hsa-miR-181b-3p	−9.8486	−2.0724	−1.7331	−2.2504
hsa-miR-181b-5p	−9.8549	−2.0786	−1.7469	−2.2459
hsa-miR-4508	−9.2885	−2.4291	−2.0458	− 2.2236
hsa-miR-215-5p	−1.8820	−2.5874	−1.5617	− 1.9192
hsa-miR-215-3p	−1.8845	−2.5652	− 1.5671	−1.9189
hsa-miR-6130	−7.1027	−3.6288	− 3.5177	−1.6216
hsa-miR-142-5p	−1.7941	−1.4349	− 1.0817	−1.4517
hsa-miR-142-3p	−1.7973	−1.4265	− 1.0828	−1.3575
hsa-miR-766-3p	−8.8234	−8.8234	−1.8474	−1.2351
hsa-miR-766-5p	−8.8202	− 8.8202	−1.8442	− 1.2319
hsa-miR-7110-5p	2.9310	1.8419	1.7966	2.4297
hsa-miR-7110-3p	2.9666	1.2266	1.8382	2.4615
hsa-miR-1-5p	5.6517	4.2842	3.9360	2.6203
hsa-miR-1-3p	5.6585	4.3807	3.8186	2.6229
hsa-miR-1246	2.0377	1.9982	2.5022	2.6648
hsa-miR-1290	1.4055	1.5543	1.9796	2.7608
hsa-miR-122-5p	1.5321	2.5392	2.5282	3.4902
hsa-miR-122-3p	1.5323	2.5394	2.5284	3.4904
hsa-miR-3591-3p	1.5433	2.5394	2.5567	3.4941
hsa-miR-3591-5p	1.5425	2.5393	2.5575	3.4942
hsa-miR-224-5p	2.1725	5.9816	6.0851	6.9059
hsa-miR-134-5p	10.0779	8.6974	7.3389	8.7835
hsa-miR-134-3p	10.0734	8.7008	7.3259	8.7969

### Target gene and functional prediction

**Fig. 2:**
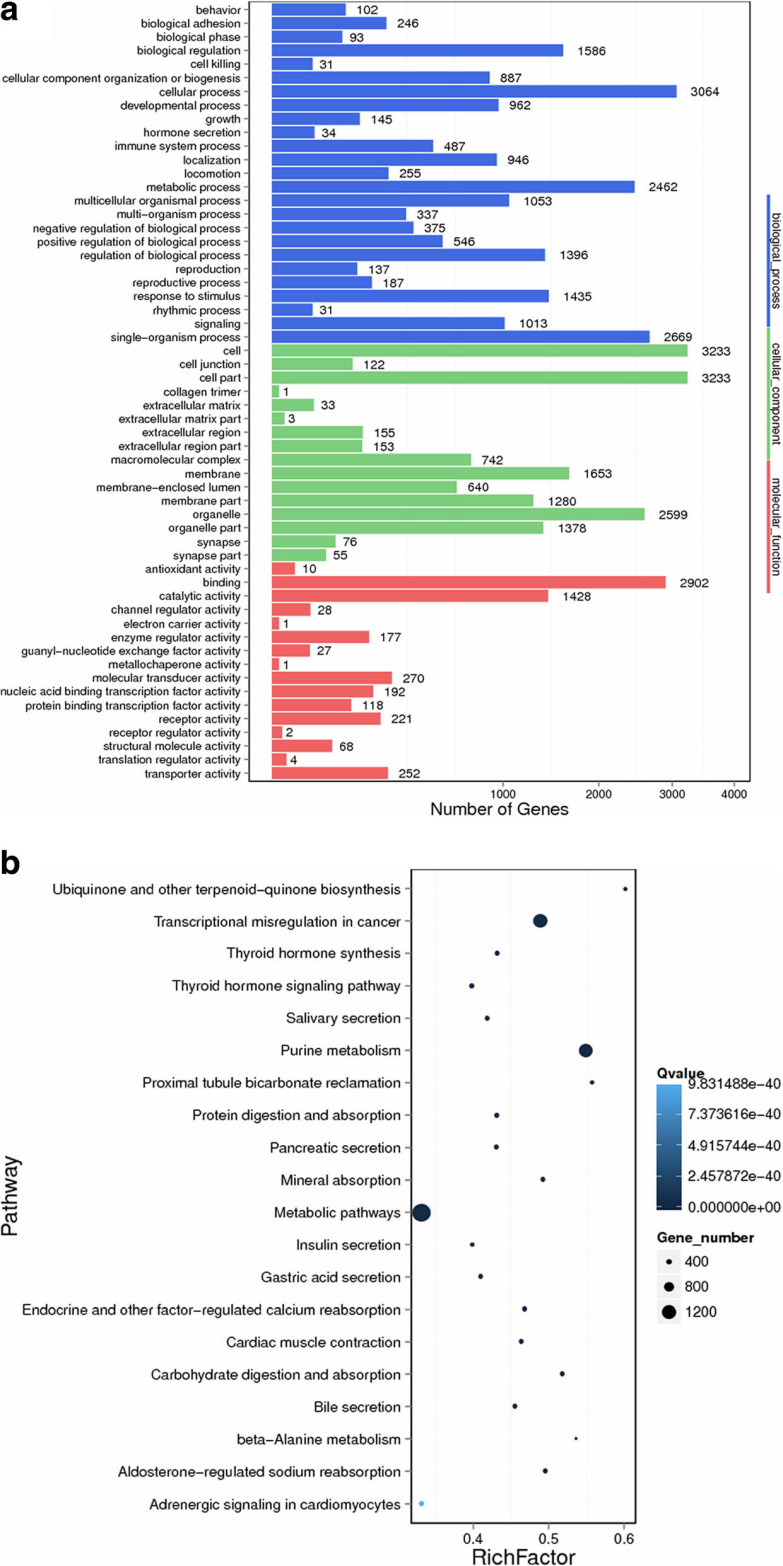
GO terms and KEGG pathways of the predicted target genes of differentially expressed miRNA. **a** The target genes of 13 upregulated and 91 coordinately downregulated miRNAs were predicted and enriched in GO terms. **b** The target genes in (**a**) were enriched for KEGG pathways

miRanda and RNAhybrid were used to predict the target of the upregulated or downregulated miRNAs. The results were adopted only when the target genes were consistent between miRanda and RNAhybrid. The functions of the target genes were enriched in Gene Ontology and KEGG. From the GO results, the biological process “cellular process” ranked first with 3064 target genes. The molecular function “binding” ranked first with 2902 target genes (Fig. 2a). However, with the KEGG pathway prediction, the largest number of targeted genes was involved in the “metabolic pathway” (Fig. 2b).

### Quantitative PCR verification of the expression levels of miRNAs

**Fig. 3:**
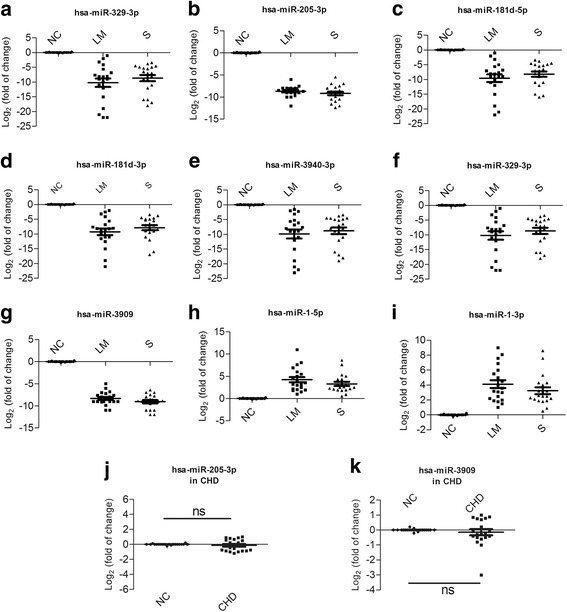
Quantitative PCR verification of 9 selected miRNAs expressed in the serum of healthy individuals and individuals with low-to-moderate (LM) and severe (S) rheumatic valvular heart disease. The results are presented as the Log_2_(fold change). The differences were statistically significant (*P* < 0.05) by the *Student t-test. "ns" represents nonsignificant*

miRNAs that have a similar downregulation or upregulation fold change in patients compared with the healthy control individual were selected for a larger cohort study by PCR quantitation. A total of 9 selected miRNAs are listed in Additional file 5: Table S5. The healthy individuals group (NC), rheumatic heart disease with low-to-moderate mitral valve stenosis group (LM) and rheumatic heart disease with severe mitral valve stenosis group (S) each contain 20 individuals. The results showed that hsa-miR-329-3p, hsa-miR-205-3p, hsa-miR-181d-5p, hsa-miR-181d-3p, hsa-miR-3940-3p, hsa-miR-136-3p and hsa-miR-3909 were significantly downregulated, with similar fold changes from RNA-sequencing (Fig. 3a-g). hsa-miR-1-5p and hsa-miR-1-3p were significantly upregulated (Fig. 3h-i). Of these 9 tested miRNAs, hsa-miR-205-3p and hsa-miR-3909 showed a low degree of dispersion between the members of each group. To further verify whether the altered expression of hsa-miR-205-3p and hsa-miR-3909 were specific to rheumatic heart disease, the expression of these two miRNAs was assessed in the serum of congenital heart disease patients. The results showed that both the hsa-miR-205-3p and hsa-miR-3909 expression levels in the serum of CHD were not significantly altered compared with healthy individuals (Fig. 3j-k). Therefore, these two miRNAs were selected for further investigation.

### Hsa-miR-205-3p and hsa-miR-3909 targeted the interleukin 1b-interleukin 1 receptor 1 pathway

**Fig. 4:**
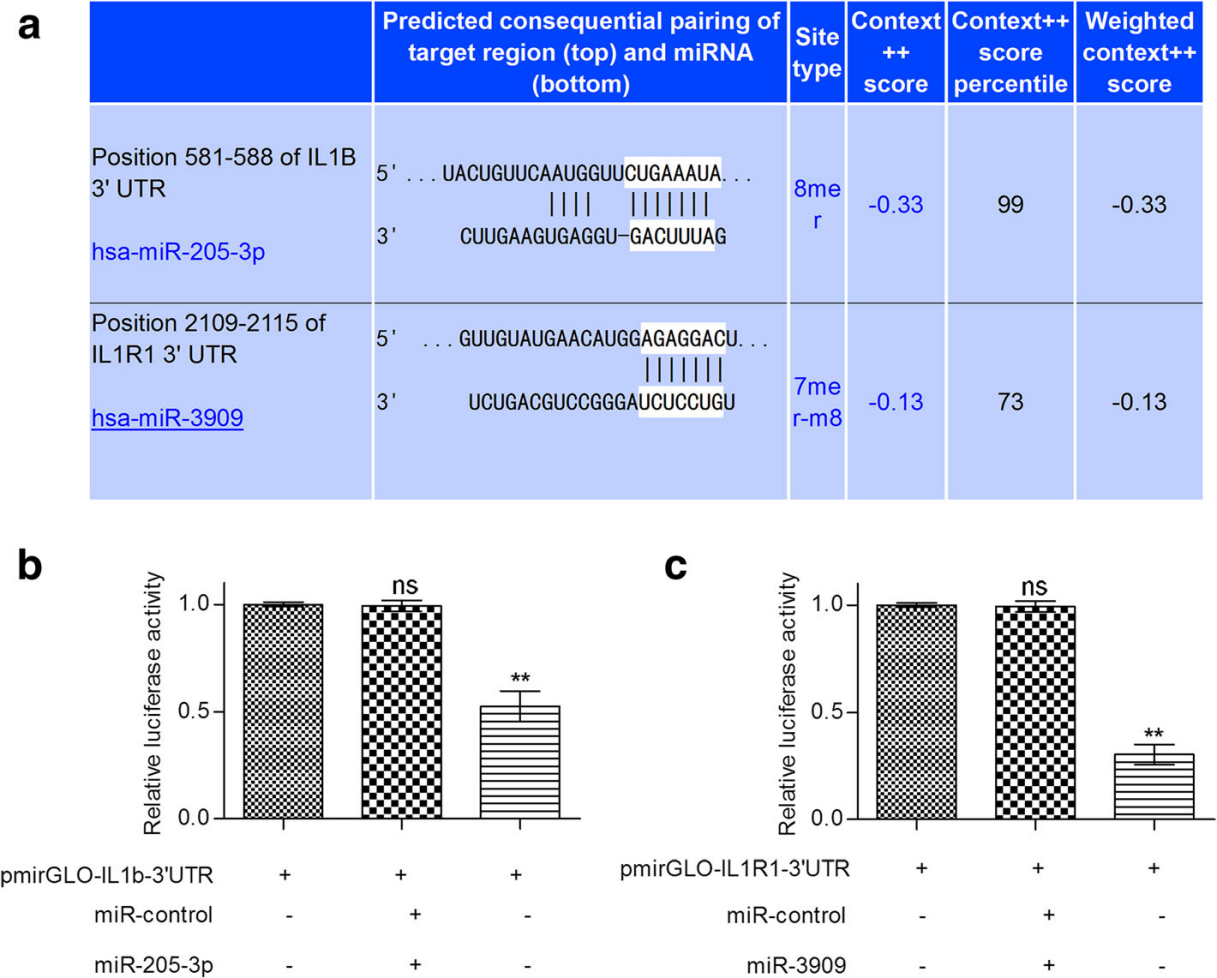
Target prediction and verification of hsa-mir-205-3p and hsa-mir-3909. **a** The 3’UTR of IL-1β and hsa-mir-205-3p were aligned, and the 3’UTR of IL1R1 and hsa-mir-3909 were aligned. The complementary sequences were marked. **b** and **c** Luciferase reporter plasmids containing the IL-1β or IL1R1 3’-UTR were co-transfected into HEK293T cells with hsa-mir-205p, hsa-3909 or control miRNA. Twenty-four hours after transfection, the luciferase activity was measured. The normalized luciferase activity in the control group was set to 1. Ns *P* > 0.05; ** *P* < 0.01 by the Student t-test

As mentioned above, we selected hsa-miR-205-3p and hsa-miR-3909 for further investigation because of their low degree of dispersion. The target of these two miRNAs was predicted by miRWalk, miRanda, RNA22 and Targetscan. Only the genes predicted by all four software programs were selected. The target genes of hsa-miR-205-3p and hsa-miR-3909 are listed in Additional file 3: Table S3. Since rheumatic heart disease is an immune-system related disease [10], we focused on the target that involved the inflammation-related genes. It was found that hsa-miR-205-3p targeted interleukin 1β (IL-1β), with a seed sequence complementary to the 3’UTR of IL-1β (Fig. 4a up). hsa-miR-3909 targeted the 3’UTR of interleukin 1 receptor 1 (IL1R1) (Fig. 4a down).

To verify the target prediction results, the 3’ UTR of IL-1β and the 3’ UTR of IL1R1 containing the target region of hsa-miR-205-3p or hsa-miR-3909 were cloned into the luciferase reporter plasmid. The plasmids were co-transfected with miRNA to observe the luciferase activity. It was shown that hsa-miR-205-3p significantly reduced the luciferase activity compared with the cells transfected with the control plasmid (Fig. 4b). Similarly, hsa-miR-3909 significantly reduced the luciferase activity of the cells transfected with the IL1R1 3’UTR containing the reporter plasmid (Fig. 4c).

### The mitral valve tissues of rheumatic heart disease showed higher IL-1β and IL1R1 levels compared with congenital heart disease

**Fig. 5:**
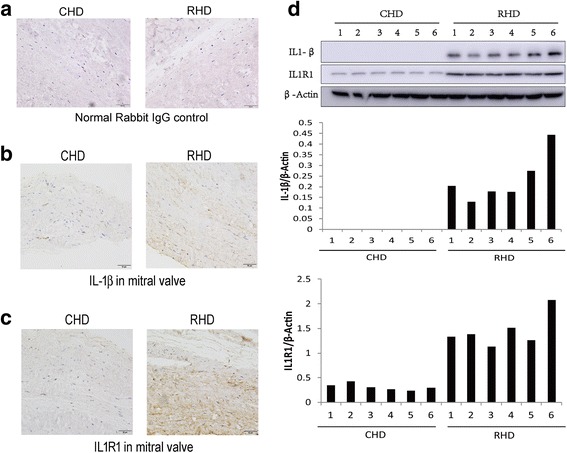
IL-1β and IL1R1 showed a higher expression level in the RHD patient mitral valve compared with the CHD patient. **a**-**c** IL-1β and IL1R1 in the mitral valve were assayed by IHC as described in the Methods using IL-1β or IL1R1 antibody **b** and **c**. Normal rabbit IgG was used to substitute antibodies as a negative control (**a**). The graph is representative of 6 CHD patients and 6 RHD patients. Bar = 50 μm. **d** The expression of IL-1β and IL1R1 was assessed by Western blot as described in the Methods section. β-actin was used as an internal control

Since hsa-miR-205-3p and hsa-miR-3909 targeted IL-1β and IL1R1, respectively, it is reasonable to infer that the downregulation of these two miRNAs may augment the expression of IL-1β and IL1R1. Mitral valve tissues were acquired by valve replacement surgery. The expression levels of IL-1β and IL1R1 were assayed by immunohistochemistry with normal rabbit IgG as a negative control (Fig. 5a). The results showed that the IL-1β expression is absent in the CHD mitral valve tissue. However, IL-1β was slightly expressed in RHD mitral valve tissue (Fig. 5b). IL1R1 was expressed in the CHD mitral valve tissue, but the expression in the RHD valve was much higher (Fig. 5c). The upregulated expression of IL-1β and IL1R1 was verified by Western blot of the mitral valve tissues from 6 CHD and 6 RHD patients (Fig. 5d).

## Discussion

Rheumatic heart disease accounts for the largest proportion of valvular disease in China, and RHD is still prevalence in developing countries [11]. Thus, it is of clinical significance to investigate its mechanism. As the best characterized non-coding RNA family, miRNAs have demonstrated a fundamental importance in normal development, differentiation and growth control and in human diseases [12]. Serum miRNA profiles generally reflect the gene expression level that influences the physiology and pathology of the body. This makes miRNAs prominent biomarkers of specific diseases and excellent therapeutic targets [13]. In the present study, using next-generation sequencing of the serum miRNA profile of a limited number of patients, the coordinately regulated miRNAs in all the patients were selected for functional investigation. It was found that two miRNAs, hsa-miR-205-3p and hsa-miR-3909, were predicted to target the IL-1β-IL-1 receptor pathway, which may upregulate the inflammation level. This was further verified by IHC of mitral valve tissues. Generally, we found that though the RHD patient experienced inflammation for more than 10 years, and the inflammation was well controlled, these patients may also have a low level of inflammation compared with patients with congenital valvular heart diseases.

Rheumatic heart disease is caused by rheumatic fever, which results from streptococcal throat infection [14]. The onset of rheumatic fever usually occurs approximately two to four weeks after a strep throat infection. Rheumatic fever results in inflammation of the heart, joints, skin or central nervous system [15]. Interleukin-1 was first described approximately three decades ago as a secreted protein of monocytes and neutrophils, referring to IL-1α and IL-1β, two key cytokines in the activation of innate immunity. The IL-1 family comprises a total of 11 members, including the two activating cytokines IL-1α and IL-1β, as well as an inhibitory mediator, the IL-1 receptor antagonist [16]. Among these, IL-1β is secreted quickly during inflammation. Generally, IL-1β acts as a potent proinflammatory cytokine at the local level, triggering vasodilatation and attracting leukocytes, including monocytes and neutrophils, to sites of infection, tissue damage and stress [17]. Furthermore, IL-1β is crucial for the induction of matrix enzymes and serves as a potent mediator of tissue damage, fibrosis and remodelling [18].

Because of the pivotal role of IL-1 signalling in rheumatic diseases, as well as rheumatoid arthritis and gouty arthritis, IL-1-targeted therapies have been successfully employed to treat a range of rheumatic diseases [19]. The role of IL-1 cytokines in acute rheumatic fever is clear [20]. However, whether the IL-1 family is involved in chronic rheumatic valvular heart disease is under investigation. It was found that interleukin-18, a member of the IL-1 family, could promote myofibroblast activation of valvular interstitial cells [21]. In the present study, we found that the aberrant miRNA profile in the serum of rheumatic valvular heart disease patients would augment the expression of both IL-1β and IL1R1, which has been proven to promote fibrosis in other tissues [18]. The comparison between congenital heart disease and rheumatic heart disease in terms of the IL-1β and IL1R1 expression levels revealed a difference between these two types of valvular heart diseases. It can be indicated that chronic inflammation may still function to promote heart valve stenosis progression even in the chronic stage with no signs of streptococcal infection. However, there are still many limitations to our present study. The downregulation of IL-1β and IL1R1 expression by the identified miRNAs was inferred from the Luciferase assay. Further verification of the involvement of IL-1β and IL1R1 includes the assessment of the expression of IL-1β and IL1R1 in the CHD and RHD mitral valves. Though we have verified the augmented expression of IL-1β and IL1R1 in RHD valve tissues in comparison with CHD valve tissues, further animal experiments investigating the potential causal role of the IL1 pathway in the progression of RHD are important.

## Conclusions

In conclusion, in the present study, we profile the serum microRNAs differentially expressed in RHD patients from healthy individuals. We point out two miRNAs, hsa-miR-205-3p and hsa-miR-3909 and their target genes IL-1β and IL1R1 are specifically involved in the progression of RHD, and suggested a potential augmentation of the IL1 pathway in rheumatic heart valve disease.

## Additional files

Additional file 1: **Table S1.** Different miRNAs expression in mild-to-moderate mitral stenosis patient LM1 and their expression fold of change compare to NC healthy control individual. (XLSX 46 kb)

Additional file 2: **Table S2.** Different miRNAs expression in mild-to-moderate mitral stenosis patient LM2 and their expression fold of change compare to NC healthy control individual. (XLSX 45 kb)

Additional file 3: **Table S3.** Different miRNAs expression in severe mitral stenosis patient S1 and their expression fold of change compare to NC healthy control individual. (XLSX 46 kb)

Additional file 4: **Table S4.** Different miRNAs expression in severe mitral stenosis patient S2 and their expression fold of change compare to NC healthy control individual. (XLSX 47 kb)

Additional file 5: **Table S5.** Nine coordinately regulated miRNAs in RHD patients and their expression fold of change compared to NC healthy control individual. (XLSX 8 kb)

## Abbreviations

CHD: Congenital heart valve disease
EF: Ejection fraction
FC: Fold change
FDR: False discovery rate
GO: Gene Ontology
IHC: Immunohistochemistry
IL1R1: Interleukin 1 receptor 1
IL-1β: Interleukin 1β
KEGG: Kyoto Encyclopedia of Genes and Genomes
LVEDD: Left ventricular end-diastolic diameter
RHD: Rheumatic heart disease
VHD: Valvular heart disease
